# Effects of Phlorotannins from *Sargassum* on In Vitro Rumen Fermentation, Microbiota and Fatty Acid Profile

**DOI:** 10.3390/ani13182854

**Published:** 2023-09-08

**Authors:** Qianqian Huang, Yuhua Chen, Xingxing Wang, Yuanhao Wei, Min Pan, Guoqi Zhao

**Affiliations:** 1 College of Animal Science and Technology, Yangzhou University, Yangzhou 225009, China; ovaltine0930@163.com (Y.C.);; 2 Institutes of Agricultural Science and Technology Development, Yangzhou University, Yangzhou 225009, China; 3 Joint International Research Laboratory of Agriculture and Agri-Product Safety, The Ministry of Education of China, Yangzhou University, Yangzhou 225009, China

**Keywords:** phlorotannins, ruminal fermentation, fatty acids, microbiota, biohydrogenation

## Abstract

**Simple Summary:**

The use of plant bioactive compounds like tannins to modulate ruminal biohydrogenation is a good strategy to optimize the fatty acid composition of ruminant-derived products, which are closely associated with human health. Differently from terrestrial tannins, there is little information on the effect of phlorotannins (PTs) from brown seaweeds on ruminal biohydrogenation. Thus, this study aimed to evaluate the effect of PT extract from *Sargassum* on in vitro rumen fermentation, fatty acid composition and bacterial community. The inclusion of PT extract had a positive effect on rumen fermentation by increasing dry matter digestibility and gas production and reducing ammonia-N concentration. Rumen biohydrogenation was profoundly inhibited by PTs as reflected in an increased unsaturated fatty acid and reduced saturated fatty acid production. The addition of PTs also changed the rumen bacterial community significantly with elevated carbohydrate-mediated bacteria. Correlation analysis found that *Prevotellaceae_UCG-001*, *Anaerovorax*, *Ruminococcus*, *Ruminobacter*, *Fibrobacter*, *Lachnospiraceae_AC2044_group* and *Clostridia_UCG-014* might be involved in the biohydrogenation process. The results suggest that the inclusion of PTs in the diet improved rumen fermentation and fatty acid composition through modulating rumen microbiota.

**Abstract:**

The fatty acid profiles of ruminant-derived products are closely associated with human health. Ruminal microbiota play a vital role in modulating rumen biohydrogenation (BH). The aim of this study was to assess the influence of dietary supplementation with phlorotannins (PTs) extracted from *Sargassum* on rumen fermentation, fatty acid composition and bacterial communities by an in vitro culture study. The inclusion of PTs in the diet increased dry matter digestibility and gas production, and reduced ammonia-N concentration and pH. PT extract inhibited rumen BH, increasing the content of *trans*-9 C18:1, *cis*-9 C18:1, *trans*-9 and *trans*-12 C18:2 and reducing C18:0 concentration. 16S rRNA sequencing revealed that PTs caused an obvious change in rumen bacterial communities. The presence of *Prevotella* decreased while carbohydrate-utilizing bacteria such as *Prevotellaceae_UCG-001*, *Ruminococcus*, *Selenomonas*, *Ruminobacter* and *Fibrobacter* increased. Correlation analysis between rumen FA composition and the bacterial microbiome revealed that *Prevotellaceae_UCG-001*, *Anaerovorax*, *Ruminococcus*, *Ruminobacter*, *Fibrobacter*, *Lachnospiraceae_AC2044_group* and *Clostridia_UCG-014* might have been involved in the BH process. In conclusion, the results suggest that the inclusion of PTs in the diet improved rumen fermentation and FA composition through modulating the rumen bacterial community.

## 1. Introduction

Compared to monogastric animal products, edible ruminant products have been associated with adverse health effects, due to their high saturated fatty acid (SFA) and low polyunsaturated fatty acid (PUFA) content, which is caused by the extensive ruminal biohydrogenation (BH) conducted by rumen microbiota [1,2]. Therefore, manipulation of ruminal BH has been attempted as way of improving the nutritional value of ruminant fat, by increasing the rumen outflow of dietary PUFA and beneficial BH intermediates, such as vaccenic acid (*trans*-11C18:1) and rumenic acid (*cis*-9, *trans*-11C18:2) [3,4].

In recent years, the use of plant bioactive compounds like tannins to modulate ruminal BH and the fatty acid (FA) composition of milk and meat [4,5,6] has attracted immediate interest from ruminant nutritionists. Tannins are a naturally occurring heterogeneous group of phenolic compounds and are widespread among terrestrial and marine plants. They are generally classified into three major groups: hydrolysable tannins (HT) and condensed tannins (CT), which are both found in terrestrial plants, and phlorotannins (PT), which are only produced by brown seaweeds [7]. The use of terrestrial tannins to manipulate ruminal fermentation has been studied extensively [8,9,10]. Its effects can be detrimental, innocuous or beneficial depending on factors such as complex chemical structure, dose ingested, basal diet and animal species [11,12]. Generally, a low-dose intake of tannins has shown beneficial effects, mainly including protection against rumen protein degradation [13], reduced ruminal methanogenesis [14] and inhibition of rumen BH [15]. In contrast to terrestrial tannins, the influence of PTs on ruminal fermentation has received far less attention. An in vitro study found PTs from *Ascophyllum nodosum* to reduce ruminal fermentation and protein degradation in a dose-dependent manner [16]. Similarly, *Laminaria digitata* PTs have also been shown to protect proteins from rumen digestion and to decrease ruminal methanogenesis [17].

The effect of terrestrial tannins on ruminal BH is one of the most extensively investigated subjects. Frutos et al. [5] reviewed the effect of tannin sources on a variety of FAs in ruminant meat and milk due to their regulatory effects on rumen lipid metabolism. Some in vitro [15,18] and in vivo [19,20] studies seem to indicate that terrestrial tannins interfere with each of the several steps in the BH process, leading to the accumulation of different intermediates. The mechanism by which terrestrial tannins inhibit ruminal BH has been related to their modulation on the rumen microbial community [21,22]. However, the effects of tannins are also dependent on several factors including their plant source, type and molecular structure. CTs and HTs exhibited different modulatory abilities on rumen microbes and biohydrogenation [23,24]. To date, there is very little information available on the effect of PTs on rumen microbiome and BH. PTs are polymers composed exclusively of phloroglucinol [25]. The hydroxyl group is the most important functional group that determines the chemical properties and biological activity of tannins. The affinity of tannins for protein and bactericidal activity may increase with the number of hydroxyl groups [11,26]. Compared to terrestrial tannins, PTs contain more hydroxyl groups and thus have stronger biological activity. PTs from *A. nodosum* has exhibited stronger antibacterial activities against *E. coli O157:H7* than terrestrial tannins from Quebracho and from *Rhus semialata* [27]. Supplementation of rumen cultures with PTs from *A. nodosum* inhibited the growth of *Fibrobacter succinogenes* and *Ruminococcus albus* and promoted the growth of *Selenomonas ruminantium*, *Ruminobacter amylophilus* and *Prevotella bryantii* [28]. Given the regulatory effect of PTs on ruminal bacteria, we hypothesized that PTs may regulate the ruminal BH of PUFAs through the regulation of rumen microbiota. Thus, a PT extract from *Sargassum* was incorporated in the base of diet to investigate its effect on ruminal fermentation, FA composition of rumen digesta and rumen bacterial community in an in vitro study.

## 2. Materials and Methods

### 2.1. Phlorotannin Extraction

Wild *Sargassum Miyabei* collected from the Yantai coastal area, China, was obtained from the peak of Rongcheng cliff on the Runyu trading line. After harvesting, the algae were rinsed with seawater to remove sand and epiphytes, and then air dried at 25 °C in a controlled environment to a moisture content of less than 5%. The air-dried whole-plant material was ground to pass a 2 mm screen. After soaking in petroleum ether overnight, 100 g of ground samples was stirred using a magnetic flee with methanol/water (8:2 *v*/*v*, 2500 mL) for 2 h at room temperature. The mixture was filtered through four layers of cheesecloth. The above steps were repeated three times and the filtrates were combined. Methanol in the filtrate was evaporated at 40 °C and the remaining aqueous fraction was freeze-dried. The concentration of PTs in the crude extract was 27.82 mg/g DM, as determined by a Folin–Ciocalteu assay [29]. 

### 2.2. In Vitro Ruminal Fermentation

The experimental animal procedures were approved by the principals of Yangzhou University, the Institutional Animal Care and Use Committee (SYXK (Su) IACUC 2012-0029). Three healthy nonlactating Holstein dairy cows (550 ± 25 kg of body weight) equipped with permanent rumen fistulas were selected as rumen fluid donors for in vitro fermentation. Diet ingredients and nutrient composition are shown in Table 1. The experimental animals were fed three times a day (8:00, 14:00 and 20:00) in Gaoyou ranch at Yangzhou University, with free access to drinking water.

The incubated substrate was rice straw/flaxseed (1:1); the flaxseed was ground in advance, and the nutritional composition of the substrate is shown in Table 2. There were two treatments: one was the control group with only the substrate, and the other was supplemented with PT extract at a concentration of 125 μg/mL incubation fluid added to the substrate. 

Rumen fluid inocula of the three dairy cows were collected via the rumen fistulas before the morning feeding and transported in prewarmed (approximately 39 °C) thermos flasks to the laboratory immediately. After straining through two layers of cheesecloth, the ruminal fluid was added to an in vitro incubation medium, consistent with that used by Onodera and Henderson [30], in a proportion of 1:2 (*v*/*v*), under constant CO_2_ flux. In each 100 mL serum bottle, 220 mg DM of substrate was incubated with 30 mL of the in vitro medium containing strained ruminal fluid. The bottles were incubated under anaerobic conditions for 24 h in an incubator set at 39.5 °C and were individually agitated every 6 h. The reaction was stopped by placing the bottles into ice water for approximately 5–10 min. At each time point of 0, 2, 4, 6, 8, 10, 12 and 24 h of culture, the long needle of the barometer was inserted into the bottle through the rubber cap to read the gas production. Once the incubation was stopped, the pH was measured in each bottle and centrifuged samples (at 976× *g* for 10 min) were collected for ammonia and volatile fatty acid (VFA) analysis.

### 2.3. Chemical Analysis

Feed samples were prepared and analyzed for DM, ash, crude protein and ether extracts according to the methods described by AOAC [31]. Neutral and acid detergent fibers (NDF and ADF) were determined using an Ankom2000 Fiber Analyzer (ANKOM Technology Corp., Macedon, NY, USA) according to the methodology supplied by the company, which is based on the methods described by Van Soest et al. [32]. NDFs were assayed with sodium sulfite and α-amylase, and both were expressed with residual ash. Incubation culture supernatants were assayed for VFA by a gas chromatograph [33] and for ammonia-N by the phenol–sodium hypochlorite colorimetric method [34]. Dry matter degradability (DMD) was calculated as the difference between the DM of the substrates before and after incubation [35].

Fatty acids were extracted and converted to methyl esters using procedures based on those described by Christie [36]. Briefly, 1 mL of incubation culture was mixed with 1.5 mL of acidified salt solution (17 mM-NaCl in 1 mM-H_2_SO_4_). An aliquot of 200 mL of triglyceride decarbonate in methanol (200 mM) was added as an internal standard, followed by 2.5 mL of methanol, and the mixture was vortexed for 1 min. Then, 2.5 mL of chloroform containing 0.2 mg/mL butylated hydroxytoluene was added and the mixture was vortexed again for 2 min. The upper layer was removed by aspiration. The lower layer was dried by passing through anhydrous sodium sulphate, and the solvent was removed by fluxing nitrogen for 20 min. The dried lipid extract was resuspended in toluene and stored at −40 °C until methylation. Dried lipid extracts were resuspended in 0.5 mL toluene, the suspension was vortexed, followed by the addition of methanolic H_2_SO_4_ (1%, *v*/*v*, H_2_SO_4_ in methanol). The tube was flushed with N_2_, then incubated at 50 °C for 1 h. After cooling, 5 mL 5% (*w*/*v*) NaCl were added and the tube was vortexed for 30 s; then, 1 mL isohexane was added and the tube was vortexed again. When layers had formed, the upper layer was transferred to a fresh tube and the isohexane extraction was repeated twice. Organic fractions were combined and 1.5 mL 2% (*w*/*v*) KHCO_3_ were added. The mixture was vortexed for 30 s and allowed to settle. The upper layer was removed, dried in a centrifugal evaporator as before and resuspended in 0.2 mL of the isohexane-butylated-hydroxytoluene solvent and transferred to a GC vial. Methyl esters were separated and quantified using a gas chromatograph (Agilent 7890A GC System, Santa Clara, CA, USA) equipped with a flame ionization detector and a 100 m fused silica capillary column (0.25 mm i.d., 0.2 μm film thickness; CP-SIL 88, CP7489, Varian Ibérica S.A., Madrid, Spain) and hydrogen as the carrier gas. The GC temperature was as follows: started at 45 °C and held for 4 min; ramped up at 13 °C/min, held for 27 min; further raised to 215 °C at a rate of 4 °C/min; and finally kept constant at 215 °C for 35 min. Analyses of all peaks were accomplished by comparing their retention time with fatty acid methyl ester standards.

### 2.4. Bacterial DNA Extraction and 16S rRNA Amplicon Sequencing

Genomic DNA was extracted from thawed incubation samples using Tiangen fecal genome extraction kit (Tiangen, Beijing, China) according to manufacturer protocols. DNA concentration and integrity were measured with ultra-microspectrophotometer (Nanodrop-1000, Thermo Fisher Scientific, Wilmington, DE, USA) and agarose gel electrophoresis. DNA was stored at −20 °C until its use for 16S rRNA amplicon sequence analyses and real-time PCR. 

V3-V4 (or V4-V5) variable regions of 16S rRNA genes were amplified with universal primers 343F (5′-TACGGRAGGCAGCAG-3′) and 798R (5′-AGGGTATCTAATCCT-3′) [37]. Amplicon quality was visualized using agarose gel electrophoresis. PCR products were purified with AMPure XP beads (Agencourt Bioscience Corporation, Beverly, MA, USA) and amplified for another round of PCR. After being purified with AMPure XP beads again, the final amplicon was quantified using Qubit dsDNA Assay Kit (Thermo Fisher Scientific, USA). Sequencing was performed on an Illumina NovaSeq 6000 with 250 bp paired-end reads (Illumina Inc., San Diego, CA, USA; OE Biotech Company; Shanghai, China). 

Library sequencing and data processing were conducted by OE biotech Co., Ltd. (Shanghai, China). Raw sequencing data were in FASTQ format. Paired-end reads were then preprocessed using Trimmomatic software (version 0.36) [38] to detect and cut off ambiguous bases (N). The software also cut off low-quality sequences with average quality score below 20 using sliding-window trimming. After trimming, paired-end reads were assembled using FLASH 1.2.11 [39]. Further denoising was performed on sequences in the following way: Reads with ambiguous, homologous sequences or below 200 bp were discarded. Reads with 75% of bases above Q20 were retained. Then, reads with chimera were detected and removed using UCHIME. These two steps were achieved using QIIME software (version 1.8.0) [40]. Clean reads were subjected to primer sequence removal and clustering to generate operational taxonomic units (OTUs) using Vsearch 2.3.4 [41] with 97% similarity cutoff. The representative read of each OTU was selected using QIIME package. All representative reads were annotated and blasted against Silva database Version 138 using RDP classifier [42] (confidence threshold was 70%).

QIIME software (version 1.8.0) was used for alpha and beta diversity analysis. The microbial diversity of samples was estimated using alpha diversity that include Chao1, Shannon-Weiner and Simpson index. The unweighted and weighted Unifrac distance matrix performed by R package was used for Principal coordinates analysis (PCoA) to estimate beta diversity.

### 2.5. Statistical Analysis

*t* test was performed to determine significant differences between fermentation parameters and FA composition of different groups using SPSS 20.0 software. R package was used to analyze the significant differences between bacterial taxa using *t* test. Correlation between ruminal FA composition and genus level microbiota was evaluated by Spearman’s correlation analysis. Standard error of the mean (SEM) was reported. For all statistical analyses, significance was declared at *p* < 0.05.

## 3. Results

### 3.1. Effect of PT Extract on In Vitro Rumen Fermentation Parameters

As shown in Table 3, the DMD and gas production rates of the substrate supplemented with PT extract were significantly higher (*p* < 0.01) than those of only substrate. The pH value and ammonia-N content of ruminal fluids incubated with the substrate supplemented with PT extract were significantly lower (*p* < 0.01) than those incubated with only substrate. The production of acetic acid and the ratio of acetic acid/propionic acid were lower (*p* < 0.05) in ruminal fluids incubated with substrate supplemented with PT extract compared with those incubated with only substrate. The presence of PT extract increased (*p* < 0.01) the production of butyric acid when compared with the control incubation fluid. There were no significant differences in the concentration of MCP and total VFA between the two groups (*p* > 0.05).

### 3.2. Effect of PT Extract on FA Composition of Fermented Ruminal Fluid

Table 4 shows the effect of PT extract on the FA profile of the fermented ruminal fluid. The supplementation of PT extract reduced the concentration of C4:0, C6:0, C18:0 (*p* < 0.01) and the concentration of C14:0 and C16:0 (*p* = 0.01) in the fermented ruminal fluid. C22:0 and *cis*-13 C22:1 were detected in the fermented ruminal fluid supplemented with PT extract, while none were detected in the control incubation fluid, which did not contain PT extract. The amount of *trans*-9 C18:1 tended to increase (*p* = 0.06) while the amount of *cis*-9 C18:1 and *trans*-9, *trans*-12 C18:2 increased significantly (*p* < 0.01) from 0.34 mg/g and 0.14 mg/g to 3.98 mg/g and 0.43 mg/g of total fatty acids in the fermented ruminal fluid supplemented with PT extract compared with the control incubation fluid. Thus, the total amount of SFAs in the fermented ruminal fluid supplemented with PT extract was significantly lower (*p* = 0.046) than that in the fermented ruminal fluid without PTs. MUFAs (*p* = 0.01) and PUFAs (*p* = 0.047) were significantly higher in the rumen culture with PTs compared to that with no PTs.

### 3.3. Effects of PT Extract on Rumen Bacterial Community

**Figure 1:**
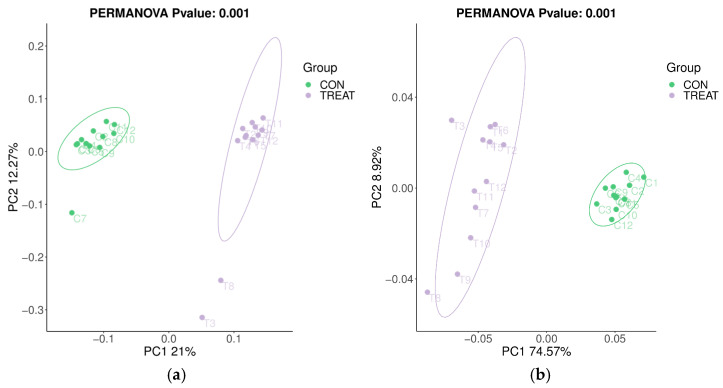
Principal coordinate analysis (PCoA) plot of the (**a**) unweighted UniFrac distances and (**b**) weighted UniFrac distances for fermented ruminal fluids. CON—control substrate; TREAT—substrate supplemented with PT extract at a concentration of 125 μg/mL incubation fluid. The percentage variation explained by each principal coordinate is indicated on the axes.

High-throughput sequencing of bacterial 16S rRNA genes yielded a total of 1,920,795 raw sequences. After quality filtering, the sequence dataset resulted in 1,235,793 high-quality sequences clustered into 5240 unique OTUs across 24 rumen samples, with an average of 51,491 sequences per sample. The diversity indices of the bacterial communities in the fermented ruminal fluid are shown in Table 5. There were no significant differences (*p* > 0.05) in the Chao1 richness index, Shannon–Weiner diversity index and Simpson index between the two fermented groups. The PCA plot of the overall rumen bacterial structure based on the unweighted and weighted UniFrac distances (Figure 1) showed that bacterial community composition clustered separately between the two fermented ruminal fluids. 

Taxonomic classification (Table 6) indicated that Bacteroidetes (68.1% on average) was the predominant phylum followed by Firmicutes (26.1% on average). The remaining bacteria phyla with an average relative abundance of over 1% were Proteobacteria (2.40%), Fibrobacteres (2.67%), and Spirochaetes (1.85%). PT extract supplementation decreased the relative abundance of Bacteroidetes (*p* < 0.01), Desulfobacterota (*p* < 0.01) and Campilobacterota (*p* = 0.04), while significantly increasing (*p* < 0.01) the relative abundance of Firmicutes, Proteobacteria, Fibrobacterota, Spirochaetota, Actinobacteriota, Patescibacteria and Elusimicrobiota. At the genus level, Prevotella, F082, Rikenellaceae_RC9_gut_group and Muribaculaceae were the predominant bacteria (Table 7); the relative abundance of Prevotella was the highest, with an average of 23.1%. The inclusion of PT extract tended (*p* = 0.06) to decrease the relative abundance of Prevotella, while significantly (*p* < 0.01) decreasing the relative abundance of F082 and Muribaculaceae, when compared with the control. The proportions of minor genera Prevotellaceae_UCG-004 (*p* = 0.02), U29-B03, Christensenellaceae_R-7_group, UCG-005, Saccharofermentans, Anaerovorax and Lachnospiraceae_UCG-008 were significantly (*p* < 0.01) reduced by the supplementation with PT extract. In contrast, significant (*p* ≤ 0.01) increases in the relative abundances of Prevotellaceae_UCG-001, Prevotellaceae_UCG-003, p-251-o5, Clostridia_UCG-014, UCG-010, Ruminococcus, Lachnospiraceae_AC2044_group, UCG-002, Selenomonas, Ruminobacter, Succinivibrionaceae_UCG-002 and Fibrobacter were observed in the fermented ruminal fluid supplemented with PT extract.

### 3.4. Correlations between Microbes and FAs in Rumen

**Figure 2:**
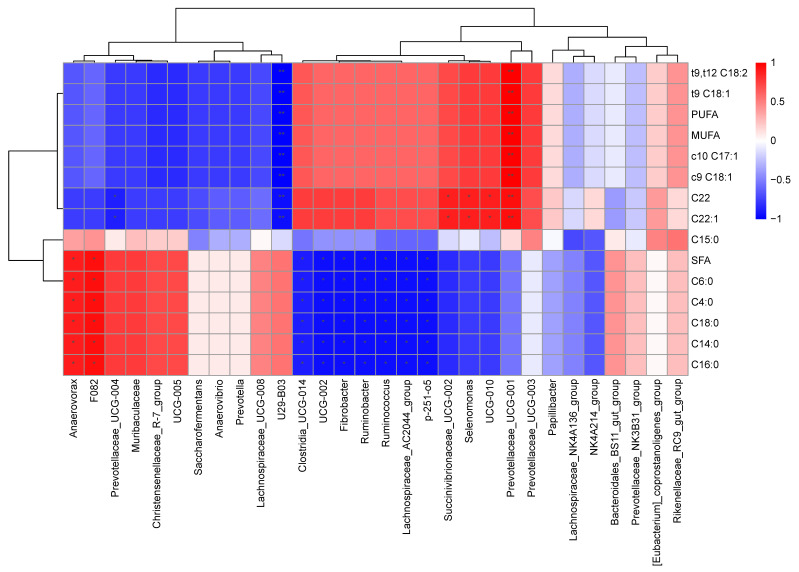
Spearman correlation analysis between fatty acid composition and the top 30 bacteria genera in fermented ruminal fluids. Significant correlations (*p* < 0.05) are indicated by *, and extremely significant correlations (*p* < 0.01) are indicated by **. Red represents positive correlation coefficients and blue represents negative correlation coefficients. The intensity of the colors represents the degree of association. The bar with numbers on the right shows the values of the correlation coefficients.

Correlations between FA composition and bacteria abundances in the rumen are shown in Figure 2. The content of *cis*-10 C17:1, *cis*-9 C18:1, *trans*-9 C18:1, *cis*-9,*trans*-9 C18:2, and total MUFAs and PUFAs correlated positively (*p* < 0.01) with abundances of Prevotellaceae_UCG-001, and negatively (*p* < 0.01) with abundances of U29-B03. The content of C22:0 and C22:1 correlated positively with the abundance of Prevotellaceae_UCG-001 (*p* < 0.01) and abundances of UCG-010, Selenomonas and Succinivibrionaceae_UCG-002 (*p* < 0.05), while correlating negatively with abundances of U29-B03 (*p* < 0.01) and Prevotellaceae_UCG-004 (*p* < 0.05). In addition, the contents of C4:0, C6:0, C14:0, C16:0, and C18:0 had positive correlations (*p* < 0.05) with abundances of F082 and Anaerovorax and negative correlations (*p* < 0.05) with abundances of Ruminobacter, Fibrobacter, UCG-002, p-251-o5, Ruminococcus, Lachnospiraceae_AC2044_group and Clostridia_UCG-014.

## 4. Discussion

### 4.1. Effects of PT Extract on In Vitro Rumen Fermentation

As with terrestrial tannins, the effect of PTs on rumen fermentation is dose-dependent. A dose–response study using batch cultures found that a purified PT extract from *A. nodosum* linearly decreased gas production and feed degradability when used at 125–500 μg/mL of rumen fluid [16]. Another study fed dried *A. nodosum* (Tasco-14TM) containing 50 g PT/kg DM (approximately 100–200 μg/mL of ruminal fluid) to lambs and cattle observed no adverse effects on feed intake or growth rate [43]. The application of PT extract from *L. digitate* at up to 40 g/kg to grass silage in vitro had no significant effects on organic matter degradation as reflected in gas production [17]. In the present study, including *Sargassum* PT extract at 125 μg/mL in the ruminal batch culture, which was equivalent to 17 g/kg DM of substrate, increased the digestion of DM and gas production, possibly because of the lower concentration used. This digestion-promoting effect might be attributed to the increase in carbohydrate-utilizing bacteria in the PT extract observed in the sequencing results, which could promote carbohydrate digestion.

A decreased ammonia-N concentration, in vitro and in the rumen, is perhaps the most common response to the inclusion of terrestrial tannins in ruminant diets [44,45]. Likewise, our study found the ammonia-N content of ruminal fluids incubated with a substrate supplemented with PT extract was reduced by almost 58%, indicating that *Sargassum* PT extract could protect dietary protein from microbial degradation and improve protein utilization in ruminants by increasing the amount of bypass protein. PT extract from *A. nodosum* and *L. digitate* also decreased rumen protein degradation in vitro [16,17]. The reduction in protein degradation in the rumen may be due to the formation of tannin–protein complexes and inhibition of the growth and activities of proteolytic bacterial populations [12].

In the present study, PT extract supplementation induced an evident shift in the VFA profile, even though the total VFA level was not affected. Acetate and acetate/propionate decreased without affecting the molar percentage of propionate. PT extract from *L. digitate* did not reduce the acetate concentration until the supplementation level rose to 40 g/kg, at which the proportion of propionate started to rise [17]. This may suggest *Sargassum* PTs possess stronger bioactivity than *L. digitate* PTs. Butyrate is often closely related to fiber degradation. Elghandour et al. [46] found that an increase in butyrate content is always accompanied by an improvement in fiber degradation ability. In this experiment, butyrate content increased significantly when PT extract was supplemented, which exactly confirmed the results of previous studies. The dramatic decrease in ammonia-N concentration and the absence of change in total VFA concentration resulted in a decrease in the pH of rumen fluid incubated with the substrate supplemented with PT extract.

### 4.2. Effects of PT Extract on Rumen FA Composition

The ability of terrestrial tannins to modulate ruminal BH is well studied. The best-characterized effect of terrestrial tannins is their inhibitory action in the last step of ruminal BH and, as a consequence, a great accumulation of intermediates, including *trans*-11 C18:1, *trans*-10 C18:1 and *cis*-9, *trans*-11-C18:2 as well as their *cis* and *trans* C18:1, C18:2 and C18:3 isomers [15,47,48]. In this study, incubation with PTs increased *cis*-9 C18:1, *trans*-9 C18:1, *trans*-9, *trans*-12 C18:2 and *cis*-13 C22:1 production while reducing C18:0 content, indicating that PTs strongly impaired the ruminal BH process. However, because of the poorly detailed FA profiles, important BH intermediates such as *trans*-11 C18:1 and *cis*-9, *trans*-*11* C18:2 were not detected. It should be mentioned that the concentration of *cis*-9 C18:1 increased substantially in response to PTs. It is difficult to determine whether the changes are due to the tannins acting in the first or last steps of BH, as they may also have been caused by the diet or by the ruminal BH of certain PUFAs. However, with the highly decreased C18:0 content as well as the low level of *cis*-9 C18:1 in the diet, we speculate that the last step from *cis* or *trans* C18:1 to C18:0 is more likely to have been suppressed.

Rumen BH is a very complex process with a multitude of steps and pathways. The key steps and effects of different sources and types of tannins in inhibiting the BH of rumen PUFAs are not consistent. Compared to HTs, CTs exerted a stronger inhibitory action on rumen BH [23,24]. CT extract from *Acacia mearnsii* inhibited the conversion of vaccenic acid to stearic acid, whereas the same concentration of sainfoin CTs reduced the hydrogenation of linolenic acid and linoleic acid but had no effect on the terminal step of BH [47]. In addition to the well-known *trans*-11 C18:1 pathway, CTs and HTs also affected the accumulations of other minor BH intermediates such as *trans*-10 C18:1 and *trans*-13 C18:1 [49,50]. In the present work, PTs may have been involved in other BH pathways, except the well-known *trans* C18:1 pathway.

### 4.3. Effects of PT Extract on Rumen Bacterial Community

Due to the process of the BH of unsaturated FAs conducted by ruminal microorganisms, high-throughput sequencing of 16S rRNA was used in this study to determine the diversity and composition of the rumen bacterial community. As far as we know, this is the first study reporting the effect of PTs on rumen microbiota when ingested by ruminants. The comparison of alpha diversity metrics revealed that the inclusion of PTs had no effect on the diversity and species abundance in the rumen bacterial community. However, clustering by treatment group was clearly observed on the PCoA plot and revealed that the structure and composition of the rumen bacterial communities was strongly influenced by the addition of PTs.

Tannins from terrestrial plants may show inhibitory or stimulatory effects on bacterial species depending on their MW and chemical structure [51,52]. It has also been demonstrated that the effect of PTs on ruminal bacteria is species-specific [16]. Similarly, in the present study, PT supplementation strongly impacted the abundance of ruminal bacteria. PTs produced substantial differences at the phylum level, which are rarely observed in other tannin-related studies. Bacteroidetes, Firmicutes and Proteobacteria were the three dominant groups of rumen bacteria independently of treatment, which is consistent with the results of previous studies [53]. According to the results, PTs seem to drive a shift from Bacteroides to Firmicutes and Proteobacteria. Firmicutes have an important function in the degradation of oligosaccharides as well as in VFA production and in the process of energy absorption [54]. A previous study found that grazing yaks with low feed efficiency showed lower Firmicutes relative abundance in the rumen [55]. The increased relative abundance of Firmicutes, which can result in a higher F/B ratio, is related to higher feed utilization in cattle [56]. In a human study, a higher F/B ratio in the gut was confirmed to be associated with obesity [57]. In this study, the PT-supplemented group exhibited a higher relative abundance of Firmicutes and F/B ratio in the rumen, indicating that the inclusion of PTs in ruminant diets has a potential capacity to increase energy utilization.

Within the Bacteroides phylum, the predominant *Prevotella* genus was significantly decreased by PTs, which use different substrates, including hemicellulose, pectin, proteins and peptides, for their growth [58]. The reduction in the *Prevotella* genus in the rumen may lead to a decrease in rumen protein degradation, which is supported by the lower ammonia-N content of ruminal fluids incubated with PTs. Terrestrial tannins, especially CTs, have also shown a strong inhibition effect against *Prevotella* [59]. The second and fourth largest genera, *F082* and *Muribaculaceae,* decreased to a larger extent than *Prevotella* and other minor genera, such as *Prevotellaceae_UCG-004* and *U29-B03* belonging to Bacteroidetes, with the inclusion of PTs; however, the functions of these genera in the rumen are unclear. Although the relative abundance of the Bacteroidetes phylum decreased, the relative abundance of *Prevotellaceae_UCG-001*, *Prevotellaceae_UCG-003* and *p-251-o5* genera within this phylum increased significantly in the PT-supplemented group. Previously, a study showed that *Prevotellaceae_UCG-001* and *Prevotellaceae_UCG-003* were positively correlated with feed efficiency [60]. They play an important role in carbohydrate degradation and VFA production [61]. The significant increase in the relative abundances of the genera *Clostridia_UCG-014*, *UCG-010*, *Ruminococcus*, *Lachnospiraceae_AC2044_group*, *UCG-002* and *Selenomonas* resulted in an increase in the Firmicutes phylum in the PT-supplemented group. The higher abundance of *Clostridia_UCG-014* might explain the lower concentration of acetate in the PT-supplemented rumen fluid, as a negative correlation of PTs with acetate in the rumen has been confirmed in a previous study [62]. *Ruminococcus* is one of the main cellulolytic bacteria in the rumen [63]. Bacteria belonging to *Lachnospiraceae* have been verifed to generate cellulase, which plays a vital role in the decomposition of fiber in the gut [64]. The relative abundances of *Ruminococcus* and *Lachnospiraceae_AC2044_group* in the PT-supplemented group were higher, which was conducive to improving fiber digestibility. *Selenomonas* are obligately saccharolytic, although some strains ferment lactate or amino acids. It has been suggested that the role of ruminal and intestinal *Selenomonas* involves the fermentation of soluble sugars and lactate in their natural environments [65]. This might be one of the reasons why DM digestion was increased by PT extract in this study. Within the Firmicutes phylum, *Christensenellaceae_R-7_group*, *UCG-005*, *Saccharofermentans*, *Anaerovorax* and *Lachnospiraceae_UCG-008* were found slightly decreased. *Saccharofermentans* in the rumen can degrade plant polysaccharides and the final fermentation products are acetate and propionate [66]. The lower abundance of *Saccharofermentans* in the PT-supplemented rumen may have produced a lower level of acetate or/and propionate, which were in line with the results of the rumen VFA profile. The genera *Ruminobacter* and *Succinivibrionaceae_UCG-002* dominated the Proteobacteria phylum in this study, and the levels of both genera increased with the inclusion of PTs. *Ruminobacter*, an amylolyic bacterium able to degrade proteins, was also found at a higher level in the rumen with dietary supplementation with *A. nodosum* or *L. digitata* [67]. A previous study showed that DMD and gas production were positively correlated with *Ruminobacter* [68], which was confirmed by the rumen fermentation data of this study. The relative abundance of *Fibrobacter*, another main cellulose-degrading bacterium in the rumen [69], was also significantly increased by the addition of PT. Our study found PTs had a positive effect on the growth of rumen cellulolytic bacteria and thus fiber degradation, which might explain the higher DMD of the PT-supplemented substrate. Conversely, terrestrial tannins have usually exhibited a profound inhibitory effect on fibrolytic bacteria and fiber digestibility [70,71]. However, higher-MW CT fractions from the *Leucaena leucocephala* hybrid decreased the relative abundance of *Ruminococcus* but increased the relative abundance of *Fibrobacter* [59]. *Ruminococcus* has also shown resistance to some specific types of tannins or polyphenols [72]. Supplementation of rumen cultures with PTs from *A. nodosum* at 500 µg/mL reduced the population of cellulolytic bacteria *F. succinogenes* but increased the populations of non-cellulolytic and total bacteria [16]. These discrepant results may indicate that PTs, similarly to terrestrial tannins, may have a selective effect on rumen bacteria depending on the dose, type, MW and chemical structure of tannins.

Due to the similar biological functions of PTs and terrestrial tannins, the mechanism by which PTs exert inhibitory effects on rumen bacteria may be associated with its binding ability with the bacterial cell wall, as well as enzyme activity inhibition, substrate deprivation and metal ion deprivation [28,73,74].

It is difficult to explain why PT supplementation increased the growth of some rumen bacteria. There are several tannin resistance mechanisms that have been suggested for rumen bacteria. The most effective and adaptive mechanisms are the formation of a thick glycoprotein that has a high binding affinity with tannins and the secretion of extracellular polysaccharides that separate the microbial cell wall from reactive tannins [73]. These bacteria may also have the ability to degrade CTs and HTs and use them as an energy source [75]; moreover, it is supposed that some of the bacteria could use PTs as an energy source, thus hydrolyzing PT to phloroglucinol [28]. We also speculate that if the addition of PTs inhibited the growth of some rumen bacteria to some extent in our study, the complex rumen environment may have favored the rapid proliferation of strains with high tolerance to PTs in the rumen ecosystem.

### 4.4. Correlation between Rumen Bacterial Community and FAs

In the last decades, bacteria in the *Butyrivibrio* group have been well-known and recognized as responsible for rumen BH [76,77,78]. Moreover, recent studies have found that other microorganisms—as-yet-uncultivated bacteria phylogenetically classified as *Prevotella* and *Lachnospiraceae incertae sedis* and unclassified *Bacteroidales*, *Clostridiales*, *Ruminococcaceae*, *Succinivibrionaceae* and *Fibrobacteriaceae*—may be involved in BH processes [79,80,81]. Terrestrial tannins affect ruminal biohydrogenation through increasing the relative abundance of *B. fibrisolvens* and decreasing the abundance of *B. proteoclasticus* [82]. Uncultured strains from the genera *Hungatella*, *Ruminococcus* and *Eubacterium* and unclassified *Lachnospiraceae* [23], *Lachnobacterium* (a genus in the *Lachnospiraceae* family) [70] as well as *Quinella*-related bacteria [83] may also play an important role in the ruminal BH process in response to dietary tannins.

In the present study, correlation results show that the content of *cis*-9 C18:1, *trans*-9 C18:1, *trans*-9, *trans*-12 C18:2 and C22:1 as well as total MUFAs and PUFAs all had a highly positive correlation with the abundance of *Prevotellaceae_UCG-001* and a strong negative correlation with the abundance of *U29-B03*, which indicates that PT supplementation increased the contents of PUFAs in the rumen by increasing the abundance of *Prevotellaceae_UCG-001* and decreasing the abundance of *U29-B03*. This also suggests that *Prevotellaceae_UCG-001* might have been involved in the initiation phase of rumen BH, while *U29-B03* might have been involved in the later steps.

The level of C18:0 in the rumen culture showed a positive relationship with abundances of *F082* and *Anaerovorax*, indicating that the large decrease in C18:0 production could be attributed to the reduction in *F082* and *Anaerovorax* induced by PT addition. *Anaerovorax*, a member of the *Lachnospiraceae incertae sedis* family, has been previously reported to be involved in the rumen BH process [79]. *Lachnospiraceae incertae sedis* might have had an involvement in the BH process when marine algae were fed as a supplement to dairy cows [78]. These results are evidence that these two genera may somehow participate in the final step of BH, the hydrogenation of C18:1 to C18:0. The negative association with abundances of *Ruminobacter*, *Fibrobacter*, *UCG-002*, *p-251-o5*, *Ruminococcus*, *Lachnospiraceae_AC2044_group* and *Clostridia_UCG-014* shows that an increase in these bacteria caused a reduction in C18:0. It is difficult to identify which step of rumen BH these genera are directly or indirectly involved in. Nevertheless, it is worth noting that many of these genera are well-known fiber-degrading bacteria such as *Ruminobacter*, *Fibrobacter* and *Ruminococcus*.

## 5. Conclusions

The addition of PT extract from *Sargassum* at 17 g/kg to in vitro substrate improved rumen fermentation as reflected by an increased dry matter degradability and gas production as well as a reduction in ammonia-N. Additionally, PTs changed the FA composition in the rumen by increasing MUFAs and PUFAs and decreasing SFAs. These effects are all closely associated with changes in the rumen bacteria community in response to PTs. PTs effectively modulated rumen microbiota by promoting the growth of carbohydrate-utilizing bacteria, such as *Prevotellaceae_UCG-001*, *Ruminococcus*, *Selenomonas*, *Ruminobacter* and *Fibrobacter*. Correlation analysis revealed that *Prevotellaceae_UCG-001*, *Anaerovorax*, *Ruminococcus*, *Ruminobacter*, *Fibrobacter*, *Lachnospiraceae_AC2044_group* and *Clostridia_UCG-014*, most of which are fiber-degrading bacteria, may play an important role in rumen BH pathways regulated by PTs. Further research is needed to confirm these effects and their associated health benefits in animal studies.

## Figures and Tables

**Table 1:** Ingredient and nutrient composition of the dairy cows’ diet (%, DM basis).

Items	Content
Ingredients	
Corn	15.96
Barley	4.71
Soybean meal	5.27
Cottonseed meal	4.34
DDGS	8.02
NaCl	0.40
Limestone	0.23
CaHPO_4_	0.34
NaHCO_3_	0.41
Premix ^1^	1.15
Oat hay	6.31
Alfalfa hay	24.62
Corn silage	28.24
Nutrient levels ^2^	
NEL/(MJ/kg)	6.26
CP	13.35
EE	3.97
NDF	40.07
ADF	23.19

^1^ One kilogram of premix contained the following: vitamin A 3,000,000 IU, vitamin D3 85,000 IU, vitamin E 1450 IU, nicotinic acid 550 mg, Cu 780 mg, Mn 930 mg, Fe 1200 mg, Zn 3600 mg, Se 21 mg, I 50 mg, Co 12 mg. ^2^ NEL was a calculated value, while other analyses were measured values.

**Table 2:** Nutrient composition and fatty acid composition of substrates (DM basis).

Items	Content
Nutrient composition, %	
DM	93.21
CP	16.78
EE	32.71
NDF	46.18
ADF	41.51
Ash	7.61
Fatty acid content, mg/g	
C4:0	0.03
C6:0	0.02
C8:0	0.04
C11:0	0.47
C12:0	0.01
C15:0	0.09
*cis*-10 C15:1	0.97
*cis*-9 C16:1	0.01
*cis*-10 C17:1	0.72
*trans*-9 C18:1	1.82
*cis*-9 C18:1	0.06
*trans*-9,*trans*-12 C18:2	0.01
*cis*-9,*cis*-12 C18:2	1.62
*cis*-6,*cis*-9,*cis*-12 C18:3	0.06
*cis*-11 C20:1	5.00
C21:0	0.02
*cis*-11,*cis*-14 C20:2	0.04
*cis*-13 C22:1	0.01
C23:0	0.01
*cis*-13,*cis*-16 C22:2	0.02

**Table 3:** Effect of phlorotannin (PT) extract on in vitro rumen fermentation parameters.

Items	CON	TREAT	SEM	*p*-Value
DMD (%)	69.81	83.81	0.81	<0.01
Gas production rate (ml/h)	38.95	56.18	1.37	<0.01
MCP (mg/mL)	0.07	0.06	0.00	0.60
Ammonia-N (mg/dL)	29.73	12.42	0.73	<0.01
pH	6.56	6.30	0.03	<0.01
Total VFA (mmol/L)	76.73	73.66	2.58	0.25
Acetate (mmol/L)	50.61	45.41	2.04	0.02
Propionate(mmol/L)	14.19	15.46	0.76	0.11
Butyrate (mmol/L)	8.28	9.85	0.25	<0.01
Acetate/Propionate	3.57	3.03	0.23	0.03

DMD—dry matter disappearance; VFA—volatile fatty acids; MCP—microbial proteins; CON—control substrate; TREAT—substrate supplemented with PT extract at a concentration of 125 μg/mL incubation fluid; SEM—standard error of the mean.

**Table 4:** Effect of phlorotannin (PT) extract on the fatty acid composition (mg/g of total fatty acids) of fermented ruminal fluid.

Fatty Acids	CON	TREAT	SEM	*p*-Value
C4:0	1.43	0.26	0.08	<0.01
C6:0	0.87	0.31	0.07	<0.01
C14:0	0.98	0.83	0.03	0.01
C15:0	0.23	0.27	0.05	0.55
C16:0	1.48	0.96	0.10	0.01
*cis*-10 C17:1	0.26	0.81	0.23	0.14
C18:0	5.48	1.86	0.23	<0.01
*trans*-9 C18:1	0.35	0.98	0.17	0.06
*cis*-9 C18:1	0.34	3.98	0.08	<0.01
*trans*-9,*trans*-12 C18:2	0.14	0.43	0.05	<0.01
C22:0	0.00	0.17	0.01	<0.01
*cis*-13 C22:1	0.00	0.23	0.08	0.10
SFA	10.47	4.65	0.40	0.046
MUFA	0.94	6.00	0.45	0.01
PUFA	0.14	0.43	0.05	0.047

CON—control substrate; TREAT—substrate supplemented with PT extract at a concentration of 125 μg/mL incubation fluid. SFA = ∑C4:0 + C6:0 + C14:0 + C15:0 + C16:0 + C18:0 + C22:0; MUFA = ∑*cis*-10 C17:1 + *trans*-9 C18:1 + *cis*-9 C18:1 + *cis*-13 C22:1; PUFA = ∑*trans*-9,*trans*-12 C18:2.

**Table 5:** Effect of phlorotannin (PT) extract on the bacterial diversity indices in fermented ruminal fluid.

Items	CON	TREAT	SEM	*p*-Value
Chao1	1319	1359	88	0.67
Shannon-Weiner	9.46	9.49	0.11	0.74
Simpson	1.00	1.00	0.00	0.92

CON—control substrate; TREAT—substrate supplemented with PT extract at a concentration of 125 μg/mL incubation fluid.

**Table 6:** Effect of phlorotannin (PT) extract on the relative abundance (%) of the top 10 rumen bacteria phyla.

Taxon	CON	TREAT	SEM	*p*-Value
Bacteroidetes	72.76	63.37	0.96	<0.01
Firmicutes	23.59	28.61	0.98	<0.01
Proteobacteria	1.85	2.95	0.14	<0.01
Fibrobacterota	0.44	3.26	0.28	<0.01
Spirochaetota	0.71	1.07	0.07	<0.01
Desulfobacterota	0.39	0.31	0.02	<0.01
Actinobacteriota	0.07	0.16	0.02	<0.01
Patescibacteria	0.04	0.12	0.01	<0.01
Elusimicrobiota	0.04	0.09	0.01	<0.01
Campilobacterota	0.05	0.02	0.01	0.04

CON—control, substrate; TREAT—substrate supplemented with PT extract at a concentration of 125 μg/mL incubation fluid.

**Table 7:** Effect of phlorotannin (PT) extract on the relative abundance (%) of the top 30 rumen bacteria genera.

Phylum	Genera	CON	TREAT	SEM	*p*-Value
Bacteroidetes	*Prevotella*	23.62	22.64	0.49	0.06
	*F082*	14.55	8.72	0.67	<0.01
	*Rikenellaceae_RC9_gut_group*	11.68	11.78	0.39	0.79
	*Muribaculaceae*	9.96	4.84	0.36	<0.01
	*Prevotellaceae_UCG-001*	2.74	3.42	0.84	<0.01
	*Prevotellaceae_UCG-003*	2.48	2.74	0.09	0.01
	*p-251-o5*	1.54	2.70	0.10	<0.01
	*Bacteroidales_BS11_gut_group*	1.34	1.27	0.10	0.49
	*Prevotellaceae_UCG-004*	0.54	0.47	0.03	0.02
	*U29-B03*	0.82	0.47	0.05	<0.01
	*Prevotellaceae_NK3B31_group*	0.73	0.66	0.06	0.26
Firmicutes	*Anaerovibrio*	4.78	4.60	0.25	0.47
	*NK4A214_group*	1.48	1.53	0.09	0.54
	*Clostridia_UCG-014*	1.21	3.48	0.29	<0.01
	*Christensenellaceae_R-7_group*	1.42	0.93	0.06	<0.01
	*UCG-005*	1.20	0.81	0.06	<0.01
	*Saccharofermentans*	1.07	0.81	0.05	<0.01
	*UCG-010*	0.95	1.51	0.06	<0.01
	*Ruminococcus*	0.95	1.49	0.08	<0.01
	*Anaerovorax*	0.85	0.57	0.03	<0.01
	*[Eubacterium]_coprostanoligenes_group*	0.79	0.78	0.06	0.90
	*Lachnospiraceae_AC2044_group*	0.62	0.88	0.05	<0.01
	*Lachnospiraceae_UCG-008*	0.58	0.43	0.03	<0.01
	*Lachnospiraceae_NK4A136_group*	0.54	0.52	0.03	0.55
	*Papillibacter*	0.50	0.55	0.04	0.25
	*UCG-002*	0.39	0.83	0.05	<0.01
	*Selenomonas*	0.20	1.92	0.28	<0.01
Proteobacteria	*Ruminobacter*	0.66	1.21	0.06	<0.01
	*Succinivibrionaceae_UCG-002*	0.58	1.04	0.07	<0.01
Fibrobacterota	*Fibrobacter*	0.44	3.25	0.28	<0.01

CON—control substrate; TREAT—substrate supplemented with PT extract at a concentration of 125 μg/mL incubation fluid.

## Data Availability

Data sharing not applicable.
